# The *Shigella* Virulence Factor IcsA Relieves N-WASP Autoinhibition by Displacing the Verprolin Homology/Cofilin/Acidic (VCA) Domain*

**DOI:** 10.1074/jbc.M116.758003

**Published:** 2016-11-23

**Authors:** Rui P. M. Mauricio, Cy M. Jeffries, Dmitri I. Svergun, Janet E. Deane

**Affiliations:** From the ‡ Cambridge Institute for Medical Research, Department of Pathology, University of Cambridge, Cambridge Biomedical Campus, Hills Road, Cambridge CB2 0XY, United Kingdom and; the § European Molecular Biology Laboratory (EMBL), Hamburg Outstation, c/o DESY, Hamburg 22067, Germany

**Keywords:** bacterial pathogenesis, fluorescence anisotropy, host-pathogen interaction, small-angle X-ray scattering (SAXS), virulence factor, actin polymerization

## Abstract

*Shigella flexneri* is a bacterial pathogen that invades cells of the gastrointestinal tract, causing severe dysentery. *Shigella* mediates intracellular motility and spreading via actin comet tail formation. This process is dependent on the surface-exposed, membrane-embedded virulence factor IcsA, which recruits the host actin regulator N-WASP. Although it is clear that *Shigella* requires N-WASP for this process, the molecular details of this interaction and the mechanism of N-WASP activation remain poorly understood. Here, we show that co-expression of full-length IcsA and the *Shigella* membrane protease IcsP yields highly pure IcsA passenger domain (residues 53–758). We show that IcsA is monomeric and describe the solution structure of the passenger domain obtained by small-angle X-ray scattering (SAXS) analysis. The SAXS-derived models suggest that IcsA has an elongated shape but, unlike most other autotransporter proteins, possesses a central kink revealing a distinctly curved structure. Pull-down experiments show direct binding of the IcsA passenger domain to both the WASP homology 1 (WH1) domain and the GTPase binding domain (GBD) of N-WASP and no binding to the verprolin homology/cofilin/acidic (VCA) region. Using fluorescence polarization experiments, we demonstrate that IcsA binding to the GBD region displaces the VCA peptide and that this effect is synergistically enhanced upon IcsA binding to the WH1 region. Additionally, domain mapping of the IcsA interaction interface reveals that different regions of IcsA bind to the WH1 and GBD domains of N-WASP. Taken together, our data support a model where IcsA and N-WASP form a tight complex releasing the N-WASP VCA domain to recruit the host cell machinery for actin tail formation.

## Introduction

*Shigella flexneri* is a Gram-negative bacterial pathogen that promotes its own engulfment by epithelial cells and gains access to the host cell cytosol by escaping the phagocytic vacuole. After replication in the cytosol, *S. flexneri* hijacks the host cell actin polymerization machinery to promote intracellular motility and cell-to-cell spread. The polymerization of actin filaments at the bacterial surface leads to the formation of comet-like actin tails, providing the driving force for bacterial propulsion (1). Actin polymerization occurs at a single pole of the bacterium and is required for disease pathogenesis (2). The 120-kDa outer membrane protein IcsA (VirG) is the sole *Shigella*-specific factor required for actin-based motility, and the intercellular spread of *Shigella* is abolished in the absence of IcsA (3–5).

**FIGURE 1.**
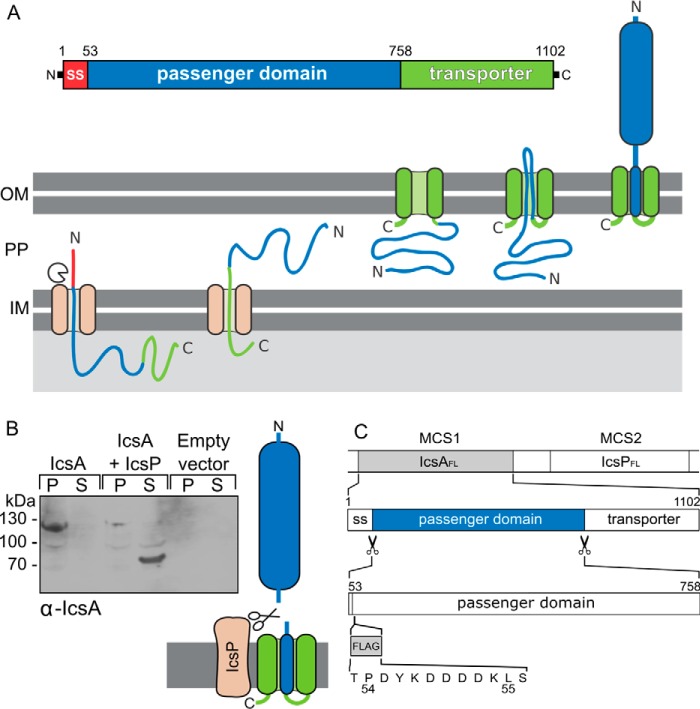
**Expression and processing of IcsA.** *A*, schematic diagram of IcsA processing and membrane insertion illustrating. The N-terminal signal sequence (*SS*, *red*) is cleaved upon translocation to the periplasm (*PP*); the passenger domain (*blue*) is exposed on the surface following translocation though the transporter domain (*green*), which is inserted in the outer membrane (*OM*). *B*, Western blot of cell pellets (*P*) and culture supernatants (*S*) following expression of full-length IcsA alone or co-expressed with IcsP. *C*, schematic diagram of the co-expression construct used for production of the soluble IcsA passenger domain. Full-length IcsA (*IcsA_FL_*) was cloned into the first multiple cloning site (MCS1) of the pETDuet-2 vector, and full-length IcsP (*IcsP_FL_*) was cloned into MCS2. Following co-expression, the signal sequence is removed, and IcsP cleaves the passenger domain from the transporter of IcsA. A FLAG tag for affinity purification was inserted into the IcsA_FL_ construct between the signal sequence and passenger domain.

IcsA is localized to the outer membrane of *Shigella* and belongs to the family of type V secreted proteins also known as autotransporters. Its transport across the bacterial inner membrane is mediated by the Sec translocon and requires an N-terminal signal peptide (residues 1–52) that is cleaved upon translocation to the periplasm (6). IcsA traverses the periplasm in complex with specific chaperones and the C-terminal transporter domain (or β-domain) (residues 759–1102) inserts into the outer bacterial membrane (7). The passenger domain (or α-domain) (residues 53–758) of IcsA is presented on the bacterial surface by translocation through the channel formed by the β-domain (6, 8) (Fig. 1*A*).

For *S. flexneri* to undergo directed movement in cells, IcsA must be localized to the pole of the bacterium. This localization is maintained, in part, by the cleavage of non-polar-localized IcsA by the outer membrane protease IcsP (9–12). IcsA is cleaved by IcsP, releasing the passenger domain from the bacterial surface (12, 13).

IcsA hijacks the host cell actin polymerization machinery by binding and activating the host actin regulator protein N-WASP (14, 15), which in turn activates the actin nucleator Arp2/3 complex (14). This interaction is specific to N-WASP because IcsA does not interact with WASP, another member of the N-WASP/WAVE family (15). N-WASP is a multidomain protein that adopts an autoinhibited conformation until activated. Autoinhibition is established by an intramolecular interaction between the C-terminal verprolin homology/cofilin/acidic (VCA)^4^ domain and the central GTPase binding domain (GBD). Activation of actin filament assembly requires release of the VCA domain for it to bind G-actin and recruit the Arp2/3 complex (14). In cells, the Rho-GTPase Cdc42 is the main regulator of N-WASP activity, by binding to the GBD to release the VCA domain (16, 17).

Binding of IcsA to N-WASP has been shown to enhance the affinity of the VCA domain for the Arp2/3 complex and of the N-terminal region (residues 1–276) for F-actin (14). This work suggested that IcsA may function in a similar manner to the activating GTPase Cdc42. However, an N-WASP mutant H208D that is incapable of binding to Cdc42 is still recruited to the *Shigella* surface and can induce actin tail formation (18–20). The molecular mechanism of N-WASP activation by IcsA remains poorly understood because regions other than the GBD have been implicated in binding to IcsA. Using N-WASP/WASP chimeric proteins, Suzuki *et al.* (15) identified that a region encompassing the N-terminal WASP-homology 1 (WH1) domain was sufficient for binding IcsA, whereas a chimeric protein incorporating N-WASP residues 159–275 (encompassing the GBD) was not capable of binding IcsA. In contrast, Lommel *et al.* (18) showed that constructs of N-WASP lacking the WH1 domain were recruited to the *Shigella* surface and capable of restoring actin tail formation although with lower efficiency and/or shorter tails. This study also showed that constructs encoding only residues 156–274 of N-WASP were capable of being recruited to the *Shigella* surface. This was in agreement with a previous study that found that residues 148–273 of N-WASP are recruited to the bacterial pole during actin polymerization (21). Interestingly, *Shigella* is still capable of inducing actin tail formation in cells expressing N-WASP missing residues 160–225, suggesting that residues 226–274 may represent a minimal recruitment domain (18). However, because residues 1–154 encompassing the WH1 domain are still capable of being recruited to the bacterial surface, there is likely to be an additional WH1 domain-mediated recruitment mechanism (18). Early work also implicated the C-terminal VCA domain of N-WASP in IcsA binding (22); however, subsequent studies have not supported the involvement of this domain (14, 15, 18).

Early work on domain mapping of IcsA showed that IcsA residues 53–508 are necessary for F-actin assembly (23), and further work showed that IcsA residues 103–433 represented the minimal region required for N-WASP binding (22). Another study confirmed the importance of IcsA residues 104–226 for N-WASP recruitment and residues 508–730 for F-actin assembly but not N-WASP interaction (24).

Here we show that the purified IcsA passenger domain is monomeric and possesses an elongated bent structure in solution. Pull-down experiments identify that the IcsA passenger domain can bind directly to both the WH1 and GBD regions of N-WASP but not the VCA domain. Binding studies show that IcsA competes with the VCA region for binding to the N-WASP GBD and that this interaction is significantly enhanced by IcsA also binding to the WH1 domain. Domain mapping of the IcsA interaction interface identifies distinct regions of IcsA that are responsible for binding to the WH1 and GBD domains, leaving the N-WASP VCA domain free to bind the Arp2/3 complex.

## Results

### 

#### 

##### The Passenger Domain of IcsA Is a Monodisperse Elongated Protein

**FIGURE 2.**
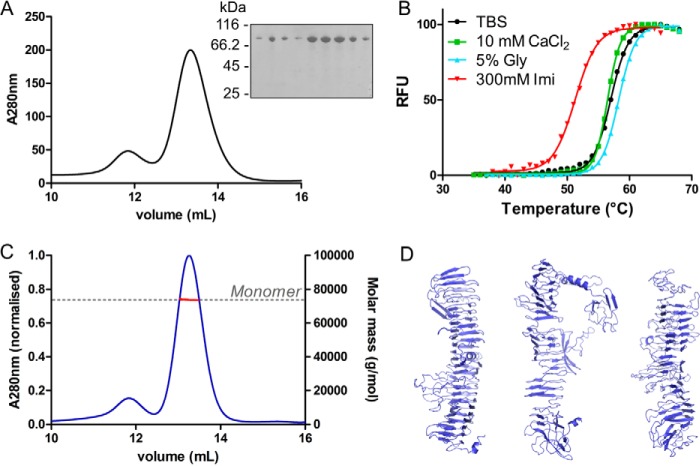
**Purification and characterization of the IcsA passenger domain.** *A*, size exclusion chromatography trace of the IcsA passenger domain purified from culture supernatant with Coomassie-stained SDS-polyacrylamide gel of peak fractions (*inset*). *B*, thermal melt curves illustrating the stabilization or destabilization of IcsA in the presence of different additives. *C*, SEC-MALS trace of IcsA passenger domain. SEC elution profiles (absorbance at 280 nm, *blue line*) and the weight-averaged molar mass shown across the elution profile (*red dots*). *D*, top three structural models of IcsA(53–758) calculated using I-TASSER. Structural alignment and matching against the PDB suggest that the protein is structurally analogous with the β-helix fold regions of hemopexin-binding protein (PDB entry 4RM6), hemoglobin protease (PDB entry 3AK5), and additional autotransporters (PDB entries 4OM9, 3SZE, 3SYJ, 3H09, and 2IOU). *RFU*, relative fluorescence units.

Expression in *Escherichia coli* of full-length IcsA including the N-terminal secretion signal and the C-terminal transporter domain followed by immunoblotting reveals a protein of ∼120 kDa present in the cell pellet consistent with membrane-bound IcsA (Fig. 1*B*). Co-expression of full-length IcsA with the *Shigella* protease IcsP releases the 72-kDa passenger domain of IcsA (IcsA(53–758)) into the culture supernatant (Fig. 1*B*). To isolate large quantities of pure IcsA(53–758), a FLAG epitope tag was inserted into this IcsA construct between the secretion signal and the N terminus of the passenger domain (Fig. 1*C*). Isolating IcsA(53–758) with FLAG antibody resin resulted in high yields of pure protein (Fig. 2*A*). Purified IcsA(53–758) was folded as shown by thermal denaturation using differential scanning fluorimetry (Fig. 2*B*). IcsA(53–758) has a melting temperature of 57 °C in Tris-buffered saline (TBS), and high throughput screening of a range of buffers, pH, and additives revealed only very minor increases in stability (*e.g.* the addition of 5% glycerol conferred a 1º C increase in the melting temperature). The addition of 300 mm imidazole was found to be destabilizing, resulting in a 6º C decrease in the melting temperature, whereas 6 m guanidinium hydrochloride unfolded IcsA, resulting in the absence of a thermal melt curve. Despite predictions that IcsA may bind calcium, the addition of 10 mm CaCl_2_ does not seem to affect the stabilization of IcsA.

Using multiangle laser light scattering coupled to size exclusion chromatography (SEC-MALS), IcsA(53–758) was shown to be monomeric with an experimentally determined molecular mass of 73 kDa (Fig. 2*C*). The early volume at which IcsA elutes off of the size exclusion column (13.3 ml) suggests that the protein may adopt an elongated structure in solution (compared with BSA at 66 kDa, which elutes at 14.2 ml). This observation is consistent with structural predictions that IcsA will possess a β-helix fold similar to that of other autotransporters. Predictive/hierarchical 3D structural modeling generated by *I-TASSER* (25–28) produces atomic models of the IcsA(53–758) passenger domain that are distinctly elongated or rodlike in nature. The top three *I-TASSER* structural predictions each possess the characteristic β-helix fold with a V-shaped cross-section (Fig. 2*D*).

**FIGURE 3.**
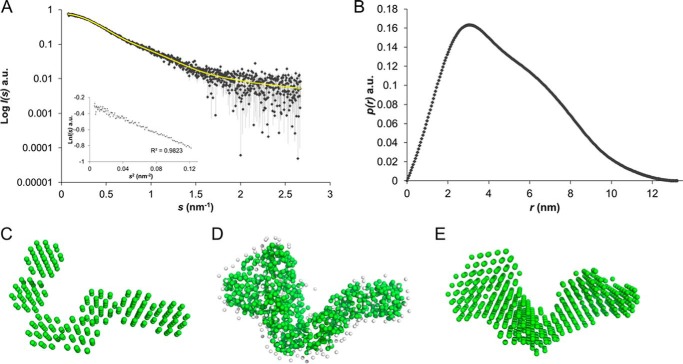
**Small angle X-ray scattering data and *ab initio* modeling.** *A*, averaged SEC-SAXS profile of IcsA(53–758) through the monomer elution (*gray diamonds*) and corresponding fit against the data of an individual *ab initio* bead model (*yellow line*). *Inset*, Guinier plot of ln *I*(*s*) *versus s*^2^ for *sR_g_* < 1.3. *B*, *p*(*r*) *versus r* profile of IcsA(53–758). *C*, *a priori* model-independent shape topology of IcsA(53–758) found using *AMBIMETER. D* and *E*, individual *GASBOR* (*D*) and *DAMMIN* (*E*) bead model reconstructions of IcsA(53–758). *a.u.*, arbitrary units.

Small-angle X-ray scattering (SAXS) was performed to obtain the primary structural parameters and overall shape characteristics of monomeric IcsA(53–758) in solution. In-line size exclusion chromatography SAXS (SEC-SAXS) was employed based on the results from SEC-MALS indicating the presence of trace higher molecular weight species in a typical IcsA(53–758) sample, thus ensuring that the scattering intensity data were measured from the separated monomeric form. In support of this, the radius of gyration (*R_g_*) through the monomer elution peak is consistent (3.5–3.8 nm) and has no significant dependence on the forward scattering intensity at zero-angle. The final averaged SAXS profile of monomeric IcsA (53–758) is shown in Fig. 3*A*, and the Guinier plot of the SAXS data is linear (*R*^2^ = 0.98; Fig. 3*A*, *inset*), as expected for aggregate-free, monodisperse systems, yielding an *R_g_* of 3.64 ± 0.02 nm. This *R_g_* is somewhat large for a protein of its size (72 kDa) (*e.g.* when compared with globular monomeric (BSA; 66 kDa) that has an *R_g_* of 2.8 nm), suggesting that IcsA(53–758) has a relatively non-globular anisotropic mass distribution. The corresponding *p*(*r*) *versus r* profile (Fig. 3*B*) supports this observation because the distribution of vector lengths is skewed, showing asymmetry for *r* > 3 nm that extends to a maximum particle dimension (*D*_max_) of ∼13 nm (BSA possesses a *D*_max_ of ∼9 nm). The *a priori* shape classification of the SAXS data places IcsA(53–758) in the “flat/extended” regime; however, both the shoulder observed on the *p*(*r*) *versus r* profile between 6 < *r* < 9 nm and the associated shape-topology from automated analysis in terms of shapes (Fig. 3*C* (29)) indicate that the protein, although structurally anisotropic, is probably bent or modular. The combined results obtained from the initial analysis of the SAXS data suggest that the predicted β-helix fold of the protein does not adopt an overall rodlike conformation typical of most autotransporters. The final structural parameters extracted from the data, including volume and molecular mass estimates, are consistent with monomeric IcsA(53–758) and the SEC-MALS results (Table 1).

**TABLE 1:** **Structural parameters for IcsA(53–758)** Molecular mass estimates from Porod volume and *datmow* are derived from scattering-invariant, *Q*, calculations (where *Q* = ∫_0_^∞^*s*^2^*I*(*s*)*ds* and *V_P_* = *2*π^2^*I*(0)/*Q*). For molecular mass Porod volume, see Ref. 48; for molecular mass *datmow* , see Ref. 50, and for molecular mass *V_c_*, see Ref. 51. In general, the dummy atom model volume/2 provides an empirical estimate of molecular mass.

*R_g_* (Guinier)	*R_g_* (*p*(*r*) *versus r*)	*D*_max_	*V_p_*, Porod volume	*V_p_*, DAM volume	MM*^a^* (Porod volume)	MM (*datmow*)	MM (*V*c)	MM (DAM)*^b^*
*nm*	*nm*	*nm*	*nm^3^*	*nm^3^*	*kDa*	*kDa*	*kDa*	*kDa*
3.64 ± 0.02	3.76 ± 0.02	13.2	103	128	64	65	67	64

*^a^* MM, molecular mass.

*^b^* Dummy atom model.

**FIGURE 4.**
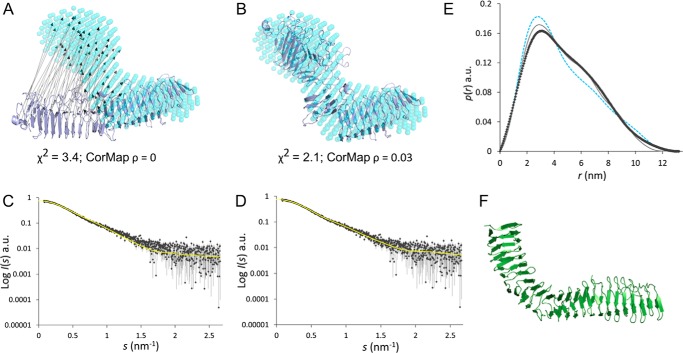
**Normal mode refinement of IcsA(53–758).** *A*, the top scoring *I-TASSER* predicted 3D atomic model (*blue ribbons*) of IcsA(53–758) undergoes bending (represented by *arrows*) during *SREFLEX* normal mode rigid body refinement against the SAXS data. *B*, final position of the post-refined atomic model of IcsA(53–758) (*cyan ribbons*). In both *A* and *B*, the pre- and postrefined atomic structures are compared with the spatially averaged bead model representation of the protein calculated independently using *DAMMIN* (*transparent cyan spheres*). *C* and *D*, the initial *I-TASSER*- and *SREFLEX*-refined IcsA(53–758) atomic model fits (yellow *lines*) to the SAXS data. *E*, a comparison between the *p*(*r*) *versus r* profiles determined from the SAXS data (*dark gray diamonds*), the initial *I-TASSER* model (*dashed blue line*), and the *SREFLEX* model (*light gray line*) showing the “hump” centered around 7 nm is caused by the bend in the IcsA structure. *F*, X-ray crystal structure of the passenger domain from Ag43a (PDB entry 4KH3). *a.u.*, arbitrary units.

Two approaches were used for *ab initio* shape analysis of IcsA(53–758): dummy atom modeling, where the shape is represented as a collection of dummy atoms (beads) using the program *DAMMIN*, and a dummy residue approach representing the protein structure by a chainlike ensemble of average residues using *GASBOR* (available on the EMBL Hamburg website). These models support the conclusions reached from the primary SAXS data analysis. The individual models representing the low resolution structure of IcsA(53–758) fit the SAXS data (χ^2^ = 1.9) with no systematic deviations observed, as assessed using Correlation Map (CorMap *p* = 0.84; Fig. 3 (*D* and *E*)) (30–32). Both the *GASBOR* and *DAMMIN* models show a characteristic ∼90° “bend” in the IcsA(53–758) monomer that occurs approximately halfway along the length of the protein so that the overall structure of the protein adopts an L-shaped conformation. When compared with the highest scoring *I-TASSER* model of IcsA(53–758), which otherwise has an almost identical *R_g_* (3.7 nm) and *D*_max_ (13 nm) as that determined from SAXS, the *I-TASSER* model does not fit the data (χ^2^ = 3.4; CorMap *p* = 0; Fig. 4*A*). The major difference between the predicted *I-TASSER* model and SAXS-based *ab initio* model is that the β-helix of the predicted structure is not discernably bent. Using an independent approach to support the conclusions reached from *ab initio* modeling, the highest scoring *I-TASSER* model underwent normal mode rigid body refinement directly against the SAXS data (*SREFLEX* (33)). It is necessary for the β-helix of the *I-TASSER* model to undergo a bend to fit the SAXS profile, giving rise to the characteristic “hump” in the *p*(*r*) *versus r* profile centered around 7 nm (Fig. 4, *A–E*; final refined model χ^2^ = 2.1; CorMap *p* = 0.03). Bent, or L-shaped, β-helices are not unprecedented for autotransporters (*e.g.* a 110° “kink” is observed approximately midway along the length of the β-helix of the antigen 43 (Ag43a) autotransporter protein from *E. coli*) (Fig. 4*F*; PDB entry 4KH3) (34).

##### IcsA Passenger Domain Binds Directly to the GBD and WH1 Domains of N-WASP

**FIGURE 5.**
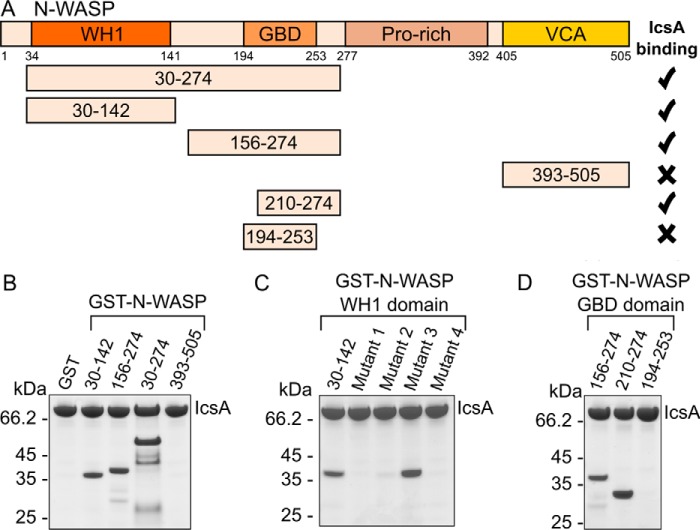
**Domain mapping of N-WASP binding to the IcsA passenger domain.** *A*, schematic diagram illustrating regions of N-WASP that bind the IcsA passenger domain. *B*, Coomassie-stained SDS-polyacrylamide gels following pull-down experiments testing binding of immobilized FLAG-IcsA (labeled) to GST-N-WASP domains. *C*, FLAG-IcsA pull-down of N-WASP/WASP chimeras encoding mutations of surface loops identified in Fig. 6*B. D*, FLAG-IcsA pull-down of N-WASP GBD truncations encoding the VCA-binding region (residues 210–274) and the Cdc42-binding region (residues 194–253).

The purified and characterized IcsA passenger domain was tested for its ability to interact with N-WASP. IcsA(53–758) bound to FLAG antibody resin was incubated with HeLa cell extracts, and isolated proteins were identified by mass spectrometry. The interaction partner N-WASP was identified in the pull-down using IcsA(53–758) (three unique peptides being identified), confirming that the expressed and purified passenger domain was functional to interact with N-WASP from human cell extracts. The N-WASP protein can be divided into four main regions: an N-terminal WH1 domain, a central GBD, a proline-rich region, and a C-terminal VCA domain. GST fusion constructs encompassing different domains of N-WASP were expressed and purified for binding studies with IcsA(53–758) (Fig. 5*A*). Pull-down experiments with FLAG-tagged IcsA(53–758) immobilized on beads identified that the WH1 domain (residues 30–142) alone and the extended GBD (residues 156–274) alone are capable of binding IcsA(53–758) (Fig. 5*B*). An extended N-WASP construct spanning the WH1 to the end of the GBD also binds IcsA but is less stable and prone to some degradation (Fig. 5*B*). The N-WASP VCA (residues 393–505) domain is not pulled down by IcsA(53–758).

**FIGURE 6.**
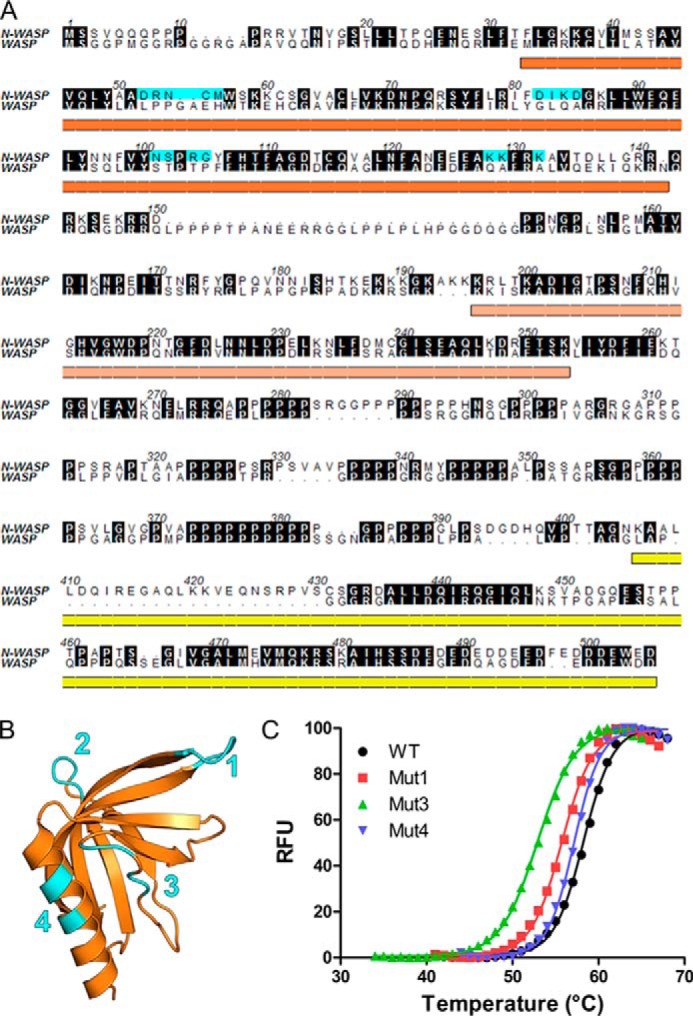
**Sequence and structural comparison of N-WASP and WASP WH1 domains.** *A*, sequence alignment of N-WASP and WASP highlighting regions of sequence divergence (*cyan*; mutant regions 1–4) and underlining domain boundaries as illustrated in Fig. 5*A. B*, structure of N-WASP WH1 domain (PDB entry 1MKE) illustrating surface patches of sequence divergence with WASP (*cyan*; mutant regions 1–4). *C*, differential scanning fluorimetry of His-tagged N-WASP/WASP WH1 chimeras. Thermal melt curves of N-WASP WH1 (*black*), mutant 1 (*red*), mutant 3 (*green*), and mutant 4 (*blue*) illustrate minimal destabilization. RFU, relative fluorescence units.

Previous work has shown that IcsA specifically binds N-WASP and not WASP (15). Sequence alignment of these two family members combined with analysis of the structure of the N-WASP WH1 (PDB entry 1MKE (35)) reveals a number of regions where the sequences diverge and that map to the surface of the protein, identifying these as potential regions where binding specificity may differ (Fig. 6, *A* and *B*). Four regions of the N-WASP WH1 domain were individually mutated to the equivalent WASP sequence: mutant 1, DRNCM → IPPGAFH; mutant 2, DIKD → GIQA; mutant 3, NSPRG → STPTP; and mutant 4, KKFRK → QAFRA. Pull-down experiments with these chimeric versions of the N-WASP WH1 domain reveal that mutations 1, 2, and 4 abrogate binding to IcsA(53–758) (Fig. 5*C*). These WH1 chimeras are not severely altered in their folding or stability (Fig. 6*C*) except for mutant 2, which, when not expressed as a GST fusion, could not be purified from *E. coli*, suggesting that this mutation has a severe detrimental effect on WH1 folding. The lack of binding to IcsA of chimeras 1 and 4 identifies these regions as determinants of the specificity of IcsA binding to N-WASP and not to WASP.

Structures of autoinhibited WASP (PDB entry 1EJ5 (36)) reveal that a short portion of the VCA domain forms a helix that binds to a region of WASP encompassing the GBD (equivalent to residues 210–274 of N-WASP). Binding of the host GTPase Cdc42 to the GBD of N-WASP destabilizes the GBD, releasing the VCA domain (17). A structure of Cdc42 bound to the GBD of WASP (PDB entry 1CEE (37)) revealed that a slightly different portion of the GBD (equivalent to residues 195–253 of N-WASP) is bound to Cdc42 and that the conformation of the GBD is vastly different upon binding to either Cdc42 or the VCA region. Pull-downs with these two shorter regions of the GBD encompassing the VCA-binding region (residues 210–274) or the Cdc42-binding region (residues 195–253) reveal binding to the VCA-binding portion but not to the Cdc42-binding portion (Fig. 5*D*). This result suggests that IcsA does not mimic the mechanism used by the normal activator Cdc42. Overall, these data identify that IcsA binds directly to two regions of N-WASP: the WH1 domain and the VCA-binding region of the GBD.

##### IcsA Competes with the VCA Peptide to Bind N-WASP and This Binding Is Enhanced by the Presence of the WH1 Domain

**FIGURE 7.**
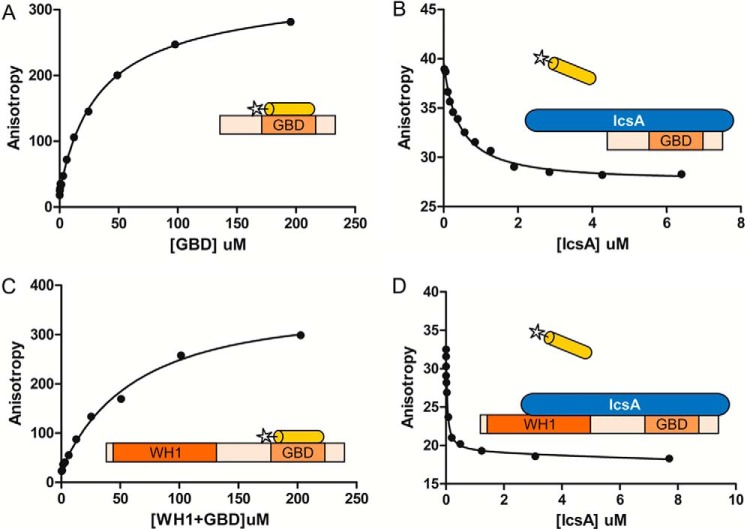
**IcsA competes with the VCA peptide for binding to N-WASP.** *A*, fluorescence anisotropy study of the binding of fluorescently labeled VCA peptide (residues 462–481; *yellow cylinder inset schematic*) to the GBD (residues 156–274). *B*, fluorescence anisotropy competition assay between IcsA and a preformed complex of GBD and fluorescently labeled VCA peptide. *C*, as for *A*, binding of fluorescently labeled VCA peptide to the WH1 + GBD (residues 30–274). *D*, as for *B*, competition assay between IcsA and a preformed complex of WH1 + GBD and fluorescently labeled VCA peptide.

To test whether IcsA binding to N-WASP may relieve autoinhibition by displacing the VCA domain, fluorescence polarization experiments were carried out using a labeled peptide encoding the region of the VCA (residues 462–481) responsible for binding to the GBD. Fluorescence polarization was monitored upon binding of this peptide to the GBD (residues 156–274), and an affinity of 28.8 ± 2.0 μm was calculated (Fig. 7*A*). Increasing concentrations of IcsA were then added to this complex, and the decrease in fluorescence anisotropy indicates release of the VCA peptide upon IcsA binding (Fig. 7*B*). IcsA efficiently competed with the VCA peptide for binding to the GBD and possessed a much higher affinity than the VCA peptide for this domain, with a *k_d_* of 0.32 ± 0.06 μm.

As shown by our pull-down experiments, the WH1 domain also contributes to binding of N-WASP to IcsA, so the longer construct of N-WASP encompassing both the GBD and WH1 domains (residues 30–274) was also used in fluorescence polarization experiments. The VCA peptide bound this construct of N-WASP with an affinity similar to that of the GBD alone, 52.4 ± 6.5 μm (Fig. 7*C*). The slightly reduced affinity of this construct when compared with the shorter GBD is probably due to the minor destabilization of this longer construct, identified previously (Fig. 5*B*), rather than to the WH1 domain interfering with binding of the GBD to the VCA peptide. Competition binding experiments revealed that IcsA was now capable of competing off the VCA peptide at a lower concentration, revealing a significantly higher binding affinity of 0.05 ± 0.01 μm (Fig. 7*D*). These data identify that binding of IcsA to the WH1 domain enhances the affinity for the GBD and allows for more efficient displacement of the VCA peptide.

##### Distinct Regions of IcsA Are Responsible for Binding the WH1 and GBD Domains of N-WASP

The elongated shape of IcsA (Fig. 4*B*) and the prediction of a β-helix type fold similar to that of other autotransporters (Fig. 2*D*) suggest that linear truncations from the N and C termini may not drastically disrupt protein folding to the extent they may with a globular protein. In support of this, the only currently available high resolution structural data for IcsA are for a portion at the C terminus of the passenger domain known as the autochaperone domain (residues 591–758) (38).

**FIGURE 8.**
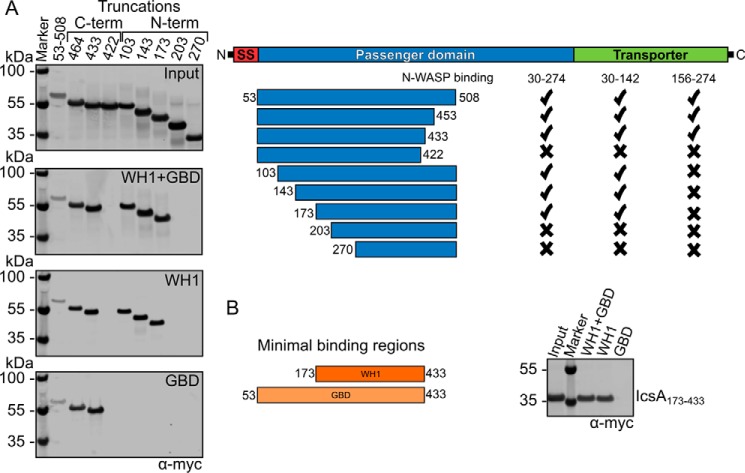
**Domain mapping of IcsA binding to the WH1 and GBD domains of N-WASP.** *A*, N- and C-terminal truncations of IcsA were produced by cell-free expression and detected by Western blotting against the Myc tag. Input gel (*top*) shows expression of all constructs and GST pull-downs against N-WASP domains; WH1 + GBD (residues 30–274), WH1 alone (residues 30–142), and GBD alone (residues 156–274) reveal different binding epitopes. *B*, the minimal binding regions for the WH1 and GBD domains are IcsA residues 173–433 and 53–433, respectively. SS, signal sequence.

The IcsA used in this study is expressed as the full-length protein and must be co-expressed in *E. coli* with IcsP. This expression system is not amenable to truncations because these modified forms of IcsA are not correctly targeted to the bacterial outer membrane, and no truncated protein can be isolated from the culture supernatant. Instead, cell-free expression systems were used to produce small quantities of Myc-tagged IcsA for pull-down experiments. A series of N- and C-terminal truncations of the IcsA passenger domain were produced in wheat germ extracts and used in pull-down experiments against N-WASP domains spanning the WH1 (residues 30–142), extended GBD (residues 156–274), and WH1 + GBD (residues 30–274) (Fig. 8*A*). The construct of IcsA encoding residues 53–508 is capable of binding all N-WASP constructs tested. This is in agreement with previous studies showing that residues 53–508 are required for F-actin assembly, and deletions within this region abolish this process (23). Upon truncation at the N terminus of the first 50 residues of the passenger domain, binding to the GBD domain is lost, but binding to the WH1 domain and the extended construct is retained. Binding to the WH1 domain is not lost until the N-terminal truncation encoding IcsA residues 203–508. Interestingly, C-terminal truncations beyond residue 433 abolish binding to all N-WASP constructs. This observation suggests that this region of IcsA may be critical for protein folding. To confirm the minimal binding region of IcsA to the WH1 domain, a construct encompassing both N- and C-terminal truncations (residues 173–433) was shown to bind to N-WASP constructs containing the WH1 but not to the GBD domain alone (Fig. 8*B*).

## Discussion

Previous studies using recombinant IcsA for interaction studies have used fusion proteins and required immunoblotting for protein detection. The quality of the protein being expressed is therefore difficult to evaluate, and interaction studies may be prone to misinterpretation due to misfolded IcsA. In this study, we have used an expression system very closely related to the native expression of IcsA by co-expressing the full-length IcsA with the membrane protease IcsP to isolate the soluble IcsA passenger domain. We have shown that this protein is correctly folded, monodisperse, and competent to bind the relevant host factor N-WASP.

Detailed analysis of SAXS data confirms that IcsA possesses an elongated shape consistent with the β-helix folds of other autotransporters. However, the SAXS data also indicate that IcsA is not a straight rod but that it possesses a central kink resulting in an L-shape, potentially similar to that of Ag43a. The kink in Ag43a is thought to help facilitate trans-mediated dimerization as part of its role as an adhesin (34). Biophysical characterization of the IcsA passenger domain shown here identifies that this domain does not dimerize, so the kink shape in this case may assist in binding to N-WASP by providing a complementary surface shape. However, a clearer understanding of the importance of this bent conformation will require structural data for the intact complex.

Using purified IcsA passenger domain, we mapped binding to regions of N-WASP. Both the WH1 and GBD domains are sufficient on their own to bind IcsA, as is a longer construct encompassing both of these domains, but the C-terminal VCA domain does not bind IcsA. The specificity for IcsA binding to N-WASP and not the related WASP protein is determined by two surface patches on the WH1 domain. Mutation of either of these patches is sufficient to abolish IcsA binding to the N-WASP WH1 domain. Another pathogen-derived protein, EspF_U_ from enterohemorrhagic *E. coli* (EHEC), that also hijacks the actin polymerization machinery of the host cell via binding to WASP family proteins, does not possess this specificity and does not bind the WH1 domain (39, 40). This observation supports our hypothesis that it is the WH1 domain that is essential for determining specificity of binding to WASP family members.

Different regions of the GBD are responsible for autoinhibition via binding to the VCA (residues 210–274) or binding the host activator protein Cdc42 (residues 194–253). These shorter fragments of the GBD were tested for binding to IcsA, and it was found that IcsA binds the short C-terminal region, which is responsible for N-WASP autoinhibition, rather than the region bound by Cdc42. Furthermore, IcsA is capable of displacing the VCA peptide from the GBD domain, suggesting that the mechanism of actin polymerization by IcsA is release of autoinhibition via displacement of the VCA domain. Displacement of the VCA from the GBD is the same mechanism used by the EHEC effector EspF_U_, indicating that despite no identifiable sequence similarity, IcsA and EspF_U_ target the same region of N-WASP for activating actin polymerization. However, unlike this EHEC effector protein, IcsA increases its affinity for N-WASP 6-fold by also binding to the WH1 domain. Thus, IcsA binding to the WH1 domain confers specificity for N-WASP and enhances the binding affinity for the GBD.

**FIGURE 9.**
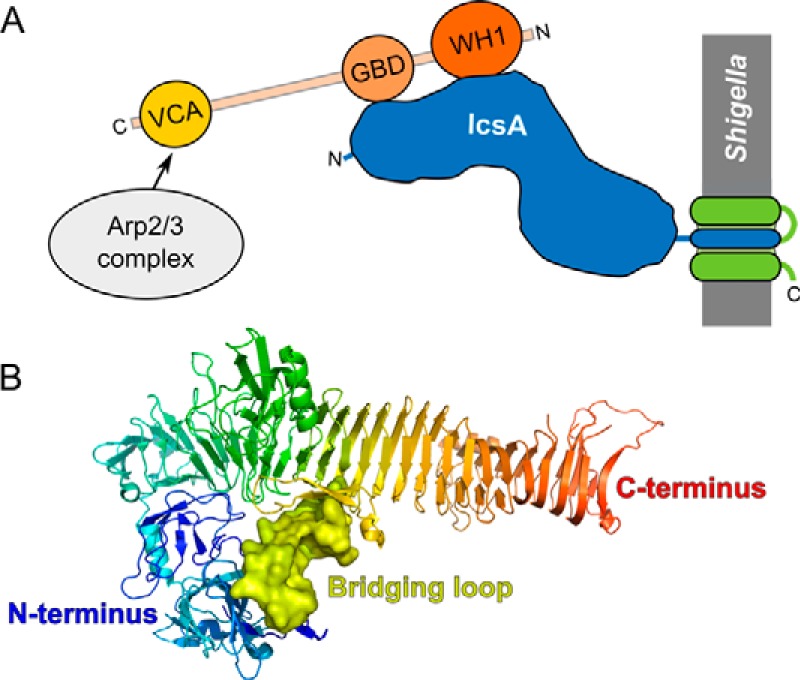
**Model of IcsA·N-WASP complex arrangement.** *A*, domain mapping of the interacting regions of the IcsA passenger domain (*blue*) with the N-WASP WH1 (*orange*) and GBD (*peach*) domains indicates that the complex will have a topology that would orient the C-terminal N-WASP VCA domain away from the *Shigella* surface, allowing it to interact with the actin polymerization machinery (Arp2/3 complex). *B*, *ribbon diagram* of the *E. coli* autotransporter, hemoglobin protease (PDB entry 1WXR, *colored blue* to *red* from the N to the C terminus), provides insight into how a central region (displayed as a surface) of a β-helix fold could stabilize the N-terminal domain.

Mapping of the regions of IcsA responsible for binding N-WASP identifies that the N-terminal WH1 domain of N-WASP binds a central region of IcsA, whereas the more C-terminal GBD region binds the N terminus of IcsA. This orientation would leave the N-WASP VCA domain available to bind the Arp2/3 complex (Fig. 9*A*). Further domain mapping of this interaction identified a region toward the C terminus of the IcsA passenger domain that may be critical for correct folding because C-terminal truncations beyond residue 433 abolished binding to all N-WASP constructs. This may be analogous to the intramolecular interactions in the autotransporter protein, hemoglobin protease, which possesses an extended loop that stretches from a central region of the protein to interact with the N-terminal domain (Fig. 9*B*; PDB entry 1WXR) (42). Loss of a bridging region like this may destabilize the fold and could explain the loss of binding to N-WASP.

In conclusion, we have identified that the IcsA passenger domain is a monomeric, elongated protein with a distinct, bent shape that specifically binds the N-WASP WH1 and GBD domains. We show that IcsA binding to the GBD domain efficiently displaces the VCA peptide and that this interaction is further enhanced by IcsA also binding to the WH1 region of N-WASP. Although release of the VCA by binding to the GBD is a mechanism used by other N-WASP activator proteins, IcsA is, as far as we know, the only activator that enhances this interaction by also binding the WH1 domain. Our results provide significant new insight into the mechanism by which the *Shigella* virulence factor IcsA binds and activates host N-WASP.

## Experimental Procedures

### 

#### 

##### DNA Constructs

Full-length *icsA* and *icsP* genes were amplified from the pWR100 virulence plasmid of *S. flexneri* (gift from Ariel Blocker), and N-WASP constructs were amplified from full-length N-WASP (gift from Bob Robinson). PCR products were amplified using KOD DNA polymerase (Novagen), cloned into pETDuet-2 (for IcsA/P co-expression), pGEX-6P-1 or pGEX-4T-2 (for GST fusions) or pF3AWG (for cell-free expression) and sequence-verified. For site-directed mutagenesis, PCR was carried out using *Pfu* Turbo® polymerase (Agilent) with primers encoding the mutation (Sigma).

##### Protein Expression and Purification

Bacterial strains used for protein expression were *E. coli* C43 (DE3) for IcsA and *E. coli* Rosetta^TM^(DE3) for N-WASP. Cultures of 2× TY medium supplemented with antibiotics were grown until an optical density (600 nm) of 0.6–0.8, when expression was induced with 1 mm isopropyl β-d-1-thiogalactopyranoside. Cultures were incubated overnight at 30 °C for IcsA or 22 °C for N-WASP. Cells were harvested by centrifugation (6,000 × *g*, 10 °C, 30 min), and pellets of N-WASP were stored frozen (−20 °C). FLAG-tagged, secreted passenger domain IcsA(53–758) was purified from medium using anti-FLAG® M2 magnetic beads. Beads were washed with TBS (50 mm Tris, pH 7.4, and 150 mm NaCl) before incubation with medium for 1 h at 4 °C on a rotating platform. The protein was eluted in TBS, 10 mm CaCl_2_ (TBSC) and 100 μg/ml 3× FLAG peptide (Sigma) (pH 7.4). Affinity chromatography was followed by size exclusion chromatography using a Superdex S200 10/300 column (GE Healthcare) pre-equilibrated in TBSC. GST-tagged N-WASP cell pellets were thawed and resuspended in TBSC with protease inhibitor (Roche Applied Science) and 400 units of DNase I (Sigma)/liter of culture. Cells were lysed using a cell disruptor (Constant Systems), and lysates were cleared by centrifugation (40,000 × *g*, 10 °C, 30 min). Proteins were purified from cleared lysate using glutathione-Sepharose 4B (GE Healthcare) washed with TBSC, 1 mm DTT and eluted with the addition of 50 mm reduced glutathione. Affinity chromatography was followed by size exclusion chromatography performed as above.

##### Sample Preparation of IcsA Expressed Alone or Co-expressed with IcsP for Immunoblot

Full-length IcsA was expressed alone or co-expressed with full-length IcsP in *E. coli* C43 (DE3) as above. 10-ml cultures were harvested by centrifugation (4,255 × *g*, 10 °C, 5 min), and pellets were diluted 1:10 in TBS. The supernatant was incubated with 10 μl of StrataClean^TM^ resin (Agilent) for 5 min, and beads were collected by centrifugation (0.5 × *g*, 2 min). Samples were boiled at 95 °C for 5 min in SDS loading dye and resolved on a 12% SDS-polyacrylamide gel followed by immunoblotting.

##### Differential Scanning Fluorimetry

4 μg of purified FLAG-tagged IcsA in TBS were combined with 1× Protein Thermal Shift Dye (Applied Biosystems) and different additives in a final volume of 20 μl before thermal denaturation was performed using an Applied Biosystems Viia7 quantitative PCR machine. Samples were heated from 25 to 95 °C at 1 °C/20 s, and fluorescence was monitored at 1 °C increments. The melting temperature (*T_m_*) was the inflection point of the sigmoidal curve obtained by curve fitting to a Boltzmann-sigmoidal model using differential scanning fluorimetry analysis scripts and Prism version 5 (GraphPad Software) (43). Melt curves for WH1 chimeras were carried out as above except in PBS.

##### MALS

Determination of molecular masses of IcsA was carried out using MALS performed at room temperature immediately following SEC on a 1260 Infinity FPLC (Agilent) by in-line measurement of static light scattering (Dawn 8+; Wyatt Technology), the differential refractive index (Optilab T-rEX; Wyatt Technology), and UV absorbance at 280 nm (Agilent). IcsA was injected onto an analytical Superdex 200 Increase 10/300 gel filtration column (GE Healthcare) in TBSC at a flow rate of 0.5 ml/min. Molecular masses were calculated using the ASTRA6 software package (Wyatt Technology).

##### I-TASSER Structural Modeling

The amino acid sequence of IcsA(53–758) was used as input for predictive/hierarchical 3D structural modeling using the Iterative Threading ASSEmbly Refinement, or *I-TASSER*, online server (25–28). Full-length structural models were generated using sequence/secondary structure chain fragment scoring against the PDB combined with multiple threading and iterative fragment assembly. Five models are by default generated and ranked using a calculated confidence score (C-score), TM score (quantitative assessment of protein structural similarity), and root mean squared S.D. against structural analogs.

##### Small Angle X-ray Scattering

Continuous flow in-line SEC-SAXS was performed at the EMBL-P12 bioSAXS beam line (PETRAIII, DESY, Hamburg) (44, 45), equipped with a Pilatus 2M detector. The SEC-SAXS data (*I*(*s*) *versus s*, where *s* = 4πsinθ/λ nm^−1^, 2θ is the scattering angle, and λ is the X-ray wavelength, 0.124 nm) were collected at room temperature using 1-s sample exposure times for a total of 3,600 1-s data frames spanning the entire course of the SEC separation monitored at 280 nm (Agilent). The IcsA(53–758) sample (75 μl at 8 mg/ml) was injected onto an analytical Superdex 200 Increase 10/300 gel filtration column equilibrated in TBSC, 3% (v/v) glycerol at a flow rate of 0.4 ml/min. The SAXS data were recorded from macromolecular free fractions corresponding to the matched solvent blank (frames 975–1,025 s) and for the separated IcsA(53–758) monomers (elution time maximum = 34.5 min, 13.6 ml; frames 2,050–2,300 s). Data reduction to produce the final scattering profile of monomeric IcsA(53–758) was performed using standard methods. Briefly, 2D-to-1D radial averaging was performed using the *SASFLOW* pipeline (46). The buffer frames spanning 975–1,025 s were checked for statistical equivalence using all pairwise comparison CorMap *p* values set at a significance threshold (α) of 0.01 (32) before being averaged to generate a final buffer scattering profile. The averaged buffer scattering was then subtracted from statistically similar data blocks collected through the IcsA(53–758) monomer elution peak. Those subtracted data blocks producing a consistent *R_g_* through the elution profile (as evaluated using the Guinier approximation (47)) were scaled and checked for similarity using CorMap before being averaged to produce the final reduced 1D scattering profile of monomeric IcsA(53–758) in solution. Primary data processing, including all CorMap calculations, was performed in PrimusQT of the ATSAS package (48). The indirect inverse Fourier transform of the SAXS data and the corresponding probable real space-scattering pair distance distribution (*p*(*r*) *versus r* profile) of IcsA(53–758) were calculated using GNOM (49), from which the *R_g_* and *D*_max_ were determined. The *p*(*r*) *versus r* profile was also used for volume and subsequent molecular weight estimates of the IcsA(53–758) monomers, as evaluated by the *datporod* (Porod volume) (48), *datmow* (50), and *datvc* (51) modules of the ATSAS 2.7.2 package. In addition, the *a priori* assessment of the non-uniqueness of the IcsA(53–758) scattering data was performed using *AMBIMETER* (29), which also generated a likely model-independent shape-topology of the protein before subsequent bead modeling. The *ab initio* bead modeling of IcsA(53–758) was performed using *GASBOR* (30) and *DAMMIN* (31). Because SAXS data can be ambiguous with respect to shape restoration (IcsA(53–758) *AMBIMETER* score = 2.8; highly ambiguous), both *GASBOR* and *DAMMIN* were run 10 times, and the consistency of the individual models was evaluated using the normalized spatial discrepancy (NSD) metric (where NSD <0.7 represents spatially similar) (52). A final averaged spatial representation of IcsA(53–758) (from *DAMMIN*; NSD = 0.7) was calculated using the *DAMAVER* set of programs (52). Normal mode rigid body refinement of the highest ranking atomic model of IcsA(53–758) predicted using *I-TASSER* was performed using *SREFLEX* (33). All individual model fits to the data were assessed using the reduced χ^2^ test as well as the CorMap *p* value (α = 0.01), the latter being independent from correct error estimation/propagation through the data reduction process (32). The SAXS data and *ab initio* bead models of IcsA(53–758) have been deposited into the Small-Angle Scattering Biological Data Bank (SASBDB) (53) under the accession code SASDB95.

##### Mass Spectrometry

Samples were resolved ∼1 cm into a precast SDS-polyacrylamide gel. The entire lane was excised and cut into four equal slices. Proteins were reduced, alkylated, and digested in-gel using trypsin. The resulting peptides were dried in a vacuum concentrator (Eppendorf) and resuspended in 8.0 μl of MS solvent (3% acetonitrile, 0.1% trifluoroacetic acid). LC-MS/MS was performed on an Orbitrap XL (Thermo Fisher Scientific) coupled to a nanoAcquity UHPLC (Waters). MS was carried out at 60,000 resolution (full width at half-maximum at *m*/*z* 400) between *m*/*z* 300 and 2,000 with MS/MS switching performed in a Top6 DDA fashion. Raw files were converted to mzXML using MSCovert (ProteoWizard) and searched against a SwissProt database with no species restrictions (545,388 entries, downloaded September 6, 2014) using Mascot version 2.3. Carbamidomethylation (C) was defined as a fixed modification, and deamidation (N/Q) and oxidation (M) were defined as potential variable modifications. Peptide and protein validation was performed in Scaffold version 4.3.

##### FLAG Pull-down

For each reaction, 50 μl of anti-FLAG bead slurry (Sigma) were added to a microcentrifuge tube and washed twice with TBS, 0.1% Nonidet P-40 (TBSN). 50 μg of purified FLAG-tagged IcsA were incubated with washed anti-FLAG® M2 magnetic beads with shaking for 10 min at 4 °C. Beads were immobilized on a magnet, and supernatant was removed before the beads were washed twice with TBSN before transferring to a flat-bottomed 96-well plate. 50 μg of GST-N-WASP in 200 μl of TBSN were incubated with the FLAG beads for 1 h at 4 °C with shaking. Beads were washed four times in TBSN with shaking for 1 min. 48 μl of TBSN supplemented with 20× FLAG peptide were added to the beads and incubated with shaking for 1 min. The eluted sample was boiled in SDS-loading dye at 95 °C for 5 min and resolved on 12% SDS-polyacrylamide gel.

##### Fluorescence Anisotropy

Fluorescence anisotropy experiments were performed at 25 °C in a Clariostar® (BMG Labtech) with an emission wavelength of 525 nm and slit width of 20 nm and with an excitation wavelength of 480 nm and slit width of 15 nm. Binding and competition experiments were performed as described previously (41). Briefly, a peptide corresponding to the interacting region of the VCA with the GBD with an N-terminal fluorescein (APTSGIVGALMEVMQKRSKA; Genscript) was dissolved in TBS, 1 mm DTT to a final concentration of 20 nm. For binding experiments, duplicate serial dilutions of GST-GBD and GST-WH1 + GBD (final concentration 0.1–200 μm) were loaded onto a black polystyrene low bind half-area 96-well plate (Corning) and mixed with the peptide (final concentration 2 nm). For competition experiments, duplicate samples of GST-GBD and GST-WH1 + GBD (final concentration 5 μm) were mixed with the peptide (final concentration 0.1 nm), followed by the addition of serially diluted IcsA (final concentration 0.01–16 μm). The anisotropy was measured continuously, and values were recorded after the readings had stabilized. Binding constants (*k_d_*) were calculated by fitting data to a one-site equilibrium binding model in Prism version 5.01 (GraphPad). Each binding experiment was repeated three times, and the mean and S.D. are reported for the three independent fits to the data.

##### Magnetic GST Pull-down with Cell-free Wheat Germ (WG) Expression

IcsA constructs for WG expression were cloned into a modified pF3A WG vector encoding an N-terminal Myc epitope tag. 3 μg of DNA were made up to a volume of 20 μl with ultrapure water and kept on ice. 30 μl of TNT® SP6 high yield wheat germ reaction mix (Promega) were added to the DNA and incubated for 2 h at 25 °C. Cell-free reactions were made up to a final volume of 200 μl with wash buffer (50 mm Tris, pH 7.4, 50 mm NaCl, 0.1% Nonidet P-40) and added to GST-N-WASP proteins (50 μg) bound to magnetic GST beads. Reactions were incubated with shaking for 1 h at 4 °C. Beads were then washed four times with wash buffer with shaking for 1 min, and 48 μl of GST elution buffer (wash buffer supplemented with 50 mm reduced glutathione) were added to the washed beads and incubated with shaking for 1 min at 4 °C. SDS loading dye was then added to the supernatant, which was boiled at 95 °C for 5 min and resolved on a 4–12% BisTris gradient gel (Sigma) and visualized by Western blotting.

##### Western Blotting

Following SDS-PAGE, proteins were transferred to a methanol-activated Immobilon-FL polyvinylidene difluoride membrane. Membranes were then blocked in 5% milk (Marvel) + PBST for 30 min followed by incubation with the primary antibody (diluted in blocking solution) overnight at 4 °C rocking. After washing, infrared fluorophore-conjugated secondary antibody was added to the membranes and incubated for 45 min. Membranes were visualized using the Odyssey infrared imager (LI-COR Biosciences).

##### Antibodies

For immunoblotting, the following were used: monoclonal mouse anti-Myc antibody clone 4A6 (Merck Millipore), polyclonal rabbit anti-IcsA (gift from Ariel Blocker), polyclonal goat IRdye800-conjugated anti-mouse IgG (LI-COR Biosciences), and polyclonal goat IRdye680-conjugated anti-rabbit IgG (LI-COR Biosciences).

## Author Contributions

J. E. D. conceived and coordinated the study. C .M. J. and D. I. S. designed, performed and analyzed the SAXS experiments. R. P. M. M. designed, performed, and analyzed all other experiments. J. E. D., R. P. M. M., D. I. S., and C. M. J. wrote the paper and prepared the figures. All authors reviewed the results and approved the final version of the manuscript.

## Footnotes

4 The abbreviations used are:

VCA: verprolin homology/cofilin/acidic
GBD: GTPase binding domain
SEC: size exclusion chromatography
MALS: multiangle laser light scattering
SAXS: small-angle X-ray scattering
EHEC: enterohemorrhagic *E. coli*
PDB: Protein Data Bank
WG: wheat germ
BisTris: bis(2-hydroxyethyl)iminotris(hydroxymethyl)methane.
